# The HOTAIR/miR-214/*ST6GAL1* crosstalk modulates colorectal cancer procession through mediating sialylated c-Met via JAK2/STAT3 cascade

**DOI:** 10.1186/s13046-019-1468-5

**Published:** 2019-11-06

**Authors:** Bing Liu, Qianqian Liu, Shimeng Pan, Yiran Huang, Yu Qi, Shuangda Li, Yang Xiao, Li Jia

**Affiliations:** College of Laboratory Medicine, Dalian Medical University, 9 Lushunnan Road Xiduan, Dalian, 116044 Liaoning Province China

**Keywords:** HOTAIR, MiR-214, *ST6GAL1*, c-Met, JAK2/STAT3 cascade

## Abstract

**Background:**

The regulatory non-coding RNAs, including long non-coding RNAs (lncRNAs) and microRNAs (miRNAs), emerge as pivotal markers during tumor progression. Abnormal sialylated glycoprotein often leads to the malignancy of colorectal cancer (CRC).

**Methods:**

Differential levels of HOTAIR and *ST6GAL1* are analyzed by qRT-PCR. Functionally, CRC cell proliferation, aggressiveness and apoptosis are measured through relevant experiments, including CCK8 assay, colony formation assay, transwell assay, western blot and flow cytometry. Dual-luciferase reporter gene assay and RIP assay confirm the direct interaction between HOTAIR and miR-214. The lung metastasis, liver metatstasis and xenografts nude mice models are established to show the in vivo effect of HOATIR.

**Results:**

Here, differential levels of HOTAIR and *ST6GAL1* are primarily observed in CRC samples and cells. Upregulated HOTAIR and *ST6GAL1* are crucial predictors for poor CRC prognosis. Altered level of *ST6GAL1* modulates CRC malignancy. Furthermore, *ST6GAL1* and HOTAIR are confirmed as the direct targets of miR-214, and *ST6GAL1* is regulated by HOTAIR via sponging miR-214. ST6GAL1 induces the elevated metabolic sialylation of c-Met, which is co-mediated by HOTAIR and miR-214. Sialylated c-Met affects the activity of JAK2/STAT3 pathway. The regulatory role of HOTAIR/miR-214/*ST6GAL1* axis also impacts CRC procession. In addition, HOTAIR mediates lung metastasis, liver metastasis and tumorigenesis in vivo. ShHOTAIR and AMG-208 are combined to inhibit tumorigenesis for successful drug development.

**Conclusion:**

The HOTAIR/miR-214/*ST6GAL1* axis commands the CRC malignancy by modifying c-Met with sialylation and activating JAK2/STAT3 pathway. Our study presents novel insights into CRC progression and provided prospective therapeutic target for CRC.

## Background

CRC is the third most common cancers, with a major burden of nearly 700,000 deaths each year worldwide [1]. Patients with localized stage live a long survival period than the patients diagnosed with distant-stage [2]. Additionally, drug resistance remains the major obstacle for CRC management. Therefore, it is urgent to identify effective markers for CRC diagnosis and clarify the potential molecular mechanism.

Glycosylation is a common type of post-translational modification of proteins. Sialylated glycoproteins are involved in cellular physiological activity and cancer development [3]. Sialyltransferases (STs) are a family of anabolic enzymes, and correlate with cancer procession [4]. Alpha 2, 6-sialyltransferases mediate the transfer of SA with an alpha 2, 6-linkage to it with terminal GAL or GALNAC residues. Abnormal *ST6GAL1* is observed in prostate cancer [5] and hepatocellular cancer [6]. ST6GAL1 restructures the sialylated glycoproteins on the cell surface, which enhances CRC malignancy [7]. ST6GAL1 is a potential enzyme to modify sialylated mesenchymal-epithelial transition factor (c-Met), hyposialylated c-Met could attenuate cell motility [8]. C-Met is a class of receptor tyrosine kinase, and exists on various epithelial cell surface [9]. C-Met functions as proto-oncogene during cancer development [10]. Specifically, CRC resistance is driven by abnormal c-Met via JAK2/STAT3 pathway [11]. However, the potential mechanism is still unstated that ST6GAL1 modulates sialylated c-Met through JAK2/STAT3 pathway during CRC malignancy.

LncRNAs are the transcripts longer than 200 nucleotides, and receive attention in cancer diagnosis and therapy [12]. LncRNAs exhibit critical roles in mediating CRC development. As an oncogenic lncRNA, HOTAIR maintains various tumor malignancies, such as gastric cancer [13] and cervical cancer [14]. HOTAIR also mediates 5-FU resistance in CRC cells [15]. MiRNAs are a class of 19–25 nucleotides transcripts, without protein-coding ability. Mechanically, miRNAs suppresses mRNA transcription by directly binding to the 3′-untranslated region (3′-UTR) [16]. Numerous studies expound that miRNAs involve in tumor progression. MiR-214 is remarkably downregulated in CRC [17] and hepatocellular carcinoma [18]. Reversed interaction between HOTAIR and miR-214 has been illustrated in ovarian cancer [19]. However, the mechanism that the HOTAIR/miR-214/*ST6GAL1* crosstalk mediates c-Met sialylation is still unknown regarding CRC malignancy.

In the present study, the differential levels and regulatory roles of *ST6GAL1* and HOTAIR are expounded. The regulatory network among HOTAIR, miR-214 and *ST6GAL1* is established, which further mediates the altered sialylation of c-Met via JAK2/STAT3 pathway. We provide a novel mechanism that the HOTAIR/ miR-214*/ST6GAL1* cross-talk modifies c-Met sialylation, and offers promising target for CRC diagnosis and therapy.

## Materials and methods

### Samples from CRC patients

The study and its informed consent have been examined and certified by the Ethics Committee of the First Affiliated Hospital of Dalian Medical University (YJ-KY-FB-2016-16). Human primary CRC and adjacent tissues were extracted from 42 patients who underwent surgical resections from June 2015 to August 2017. The tumors were identified different stages (stage I, II, III and IV) based on the histopathological assessment. The samples were stored in liquid nitrogen for further analysis.

### CRC cell culture

The CRC cell lines SW620, SW480, HCT-8, HCT-8/5-FU and LoVo were purchased from Keygen Biotech Co. Ltd. (Nanjing, China). SW620 and SW480 were cultured in Leibovitz’s L-15 (Gibco, Grand Island, NY) medium contained 10% inactivated fetal bovine serum (Gibco, Grand Island, NY). HCT-8, HCT-8/5-FU and LoVo cells were cultured in in RPMI 1640 (Gibco, Grand Island, NY) medium contained 10% inactivated fetal bovine serum. To develop the 5-FU resistant LoVo cells, 5-FU was added to the medium by stepwise increasing concentrations for over 6 months. HCT-8/5-FU and LoVo/5-FU were maintained in medium supplemented with 124.5 μM and 113.0 μM 5-FU. CRC cells were both incubated at 37 °C with a humidified atmosphere containing 5% CO_2_. All cells lines were routinely tested for mycoplasma, which were shown to be negative.

### Real-time PCR analysis

The total RNA was extracted by Trizol (Invitrogen), and cDNA was synthesized by QuantiTect ReverseTranscription Kit (Qiagen, Valencia, CA). qRT-PCR was performed under an ABI Prism7500 fast real-time PCR system (Applied Biosystems, Foster City, CA) with mixing a QuantiTect SYBR Green PCR Kit (Qiagen, Valencia, CA). Relative RNA expression was calculated with normalization to GAPDH.

### Fluorescence in situ hybridization (FISH)

Cells were and fixed with 4% paraformaldehyde and dehydrated. Cells were hybridized with biotin-labeled HOTAIR and miR-214 probes (GenePharma) at 50 nM for at 73 °C for 5 min. The cells were degenerated and incubated at 37 °C overnight. Images were shown under a fluorescence microscope.

### Western blot analysis

Proteins were electrophoresed with 10% SDS-PAGE gels and transferred to the polyvinylidene difluoride membranes (Millipore, Bedford, MA, USA). Primary antibodies were incubated at 4 °C. The membrane was incubated with anti-rabbit IgG at 37 °C. All bands were determined and analyzed by LabWorks (TM ver4.6, UVP, BioImaging Systems, NY, USA). GAPDH was used as control.

### Lectin pull-down assay

The CRC cells were lysed and treated with *Sambucus nigra* lectin (SNA, Vector Laboratories, Burlingame, CA) at 4 °C overnight. Glycoprotein eluting solution was used to wash the precipitated lysates. The relevant glycoproteins were collected and analyzed by western blot.

### Cell transfection

The ampliations of *ST6GAL1* and HOTAIR were cloned into pmirGLO vector (Promega). MiR-214 mimic, inhibitor, miR-NC, short hairpin RNA for HOTAIR and scramble shRNA were synthesized by GenePharma. Lipofectamine 3000 (Invitrogen, Carlsbad, CA, USA) was used for the transfection assay. qRT-PCR was used to detect the transfected efficiency.

### Dual luciferase reporter gene assay

The pmirGLO Dual-Luciferase miRNA Target Expression Vector was obtained from GenePharma. The mRNA expression was shown by firefly luciferase, and the renilla luciferase intensity was used as normal control. Lipofectamine 3000 was utilized for the co-transfection. The dual luciferase reporter assay system (Promega) was used to conduct the experiment. Each experiment was performed in triplicate.

### Cell viability assay

Cell proliferation assay was conducted by using cell counting kit-8 (CCK-8; Dojindo, Japan). 1 × 10^3^ cells were plated into 96-well plate and 11 μL CCK8 was added into the plate for 4 h. The spectrometric absorbance was measured by microplate reader (Model 680; Bio-199 Rad, Hercules, CA, USA) at 450 nm. For chemoresistance to 5-FU, corresponding 5-FU was added into 96-well plate. Similarly, the absorbance was then measured to evaluate the chemoresistance to 5-FU. Each experiment was performed thrice.

### Focus formation assay

2 × 10^3^ single-cell suspension was obtained and seeded in cell culture dishes. The medium was renewed at regular intervals. The foci were formed obviously 12 days later. The colonies were fixed by 4% paraformaldehyde for 20 min, and stained with 0.2% crystal violet. The colonies were recorded and counted.

### Transwell assay

Cells were cultured in Boyden chambers containing a transwell membrane filter (Corning, New York, USA), and in serum-free medium overnight. The filter was coated with gelatin and matrigel. 5 × 10^4^ cells were suspended on top, and complete medium was placed in the lower. The upper side cells were removed by a cotton swab. The invading cells were counted to estimate the invasive capacity. Five random fields were analyzed for each chamber.

### Immunoprecipitation

The immunoprecipitation was performed by the protein A/G agarose beads (Thermo Fisher Scientific). Cell lysates incubated in immunoprecipitation lysis buffer (Beyotime) for 10 min. The lysates were conjugated with anti-c-Met (3 μg/ml, Abcam). The immunoprecipitates were washed with protein A/G agarose beads and used for further experiment.

### Flow cytometry

Cells were incubated with FITC-SNA (Vector Laboratories, Burlingame, CA, USA) for 1 h. The FITC fluorescence intensity was detected by FACS Calibur (Becton-Dickinson, CA, USA). CRC cells were treated with 5-FU for 48 h and resuspended in 100 μL binding buffer. Annexin V and propidium iodide were used to stain cells for 10 min. With the usage of Annexin-V-FITC apoptosis detection kit (BD, Franklin Lakes, NJ, USA), the apoptotic cells were detected by FACS Calibur.

### Immunohistochemistry (IHC) staining

The samples were collected and embedded with paraffin. The section was pre-treated with drying, deparaffining and rehydrating. The slides were immersed with 3% hydrogen peroxide for 10 min and labeled with the corresponding antibodies at 4 °C overnight. The secondary streptavidin-horseradish peroxidase conjugated antibody was used to label the primary antibody for 1 h. The slides were then counter stained with hematoxylin for 30s and cover slipped.

### RNA immunoprecipitation (RIP) assay

The endogenous miR-214, combined with HOTAIR, was pulled down. The cell lysis was incubated in RIP immunoprecipitation buffer containing magnetic bead, conjugated with human anti-Ago2 antibody (Millipore). Mouse IgG (Millipore) was used as negative control. The protein was digested by proteinase K, and the immunoprecipitated RNA was obtained. The qRT-PCR assay was conducted to detect the isolated RNAs.

### Tumor xenograft models

The experiments were approved by the Committee on the Ethics of Animal Experiments of the Dalian Medical University. The 4-week-old male nude mice were obtained from the Model Animal Research Institute of Nanjing University.

The lung metastasis model was built by injecting 5 × 10^6^ cells into the tail vein. The procession was sustained at least 10 min. The mice were then sacrificed, and the lung was isolated.

For liver metastasis, 4-week-old male nude mice were randomly used to build the liver metastasis model under anesthetizing. The spleen was exposed and injected with 5 × 10^6^ CRC cell, and this procession sustained at least 5 min. The endpoints of in vivo metastasis experiments were based on the presence of clinical signs of liver metastasis, weight loss, the appearance of ascites and energielos with reduced action. The nude mice were sacrificed 28 days later, and the liver and the spleen were collected and photographed.

For xenografts model, 1 × 10^7^ GFP labeled CRC cells were injected subcutaneously into the right flank of each nude mouse. The mice were randomly divided into control and treatment groups when the mice bearing palpable tumors. The groups received DMSO or AMG-208, respectively. The endpoints of in vivo xenografts experiments were based on the presence of weight loss, tumor volume higher than 4cm^3^ and energielos with reduced action. The representative X-Ray photos and fluorescent photos were captured to reveal the tumor volume in vivo. The photos were taken by in-vivo Imaging System of Dalian Medical University. Then the mice were humanely sacrificed and the tumors were isolated for further analysis.

### Statistical analysis

SPSS 17.0 software was used to analyze the experimental data. Data were presented as means ± standard deviation (SD), and each experiment was carried out thrice at least. Student’s t-test was used to compare the significant difference of two groups. SD represented the variation of data values. The data variance of each group was similar. The one-way analysis of variance (ANOVA) was used to determine the significant difference of multiple groups. The survival curves were calculated by Kaplan-Meier method, and the difference was assessed by a log-rank test. Spearman’s correlation analysis was used to identify the association between miRNAs and mRNA expression. Statistical significance was defined as *P* value < 0.05.

## Results

### HOTAIR and ST6GAL1 are upregulated during CRC progression

**Fig. 1:**
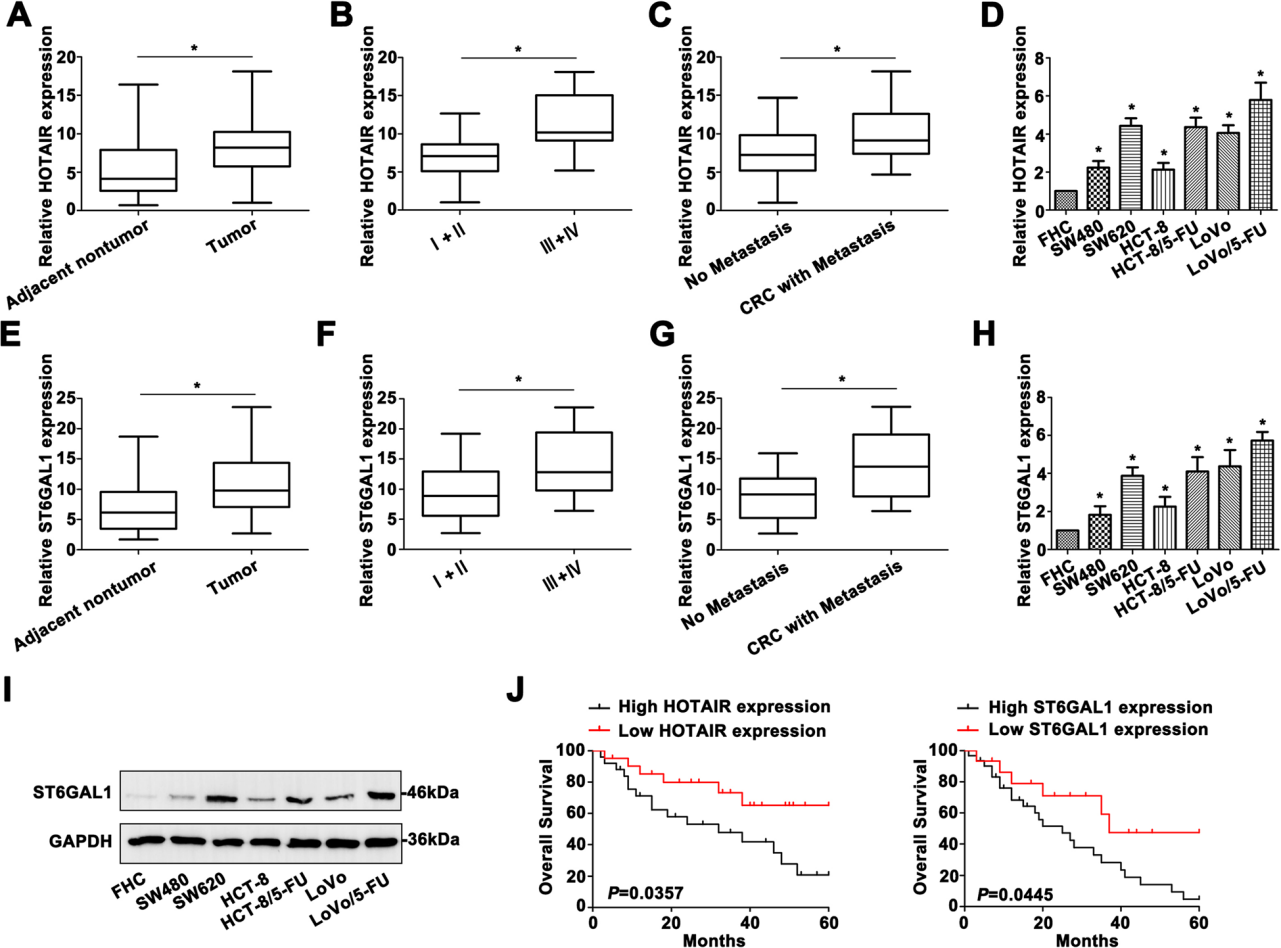
HOTAIR and ST6GAL1 are upregulated during CRC progression. **a** Differential HOTAIR expression was detected between CRC tissues and the corresponding non-tumor tissues. **b** High HOTAIR level was determined in advanced CRC stages. **c** Overexpressed HOTAIR was identified in CRC patients with metastasis. **d** HOTAIR expression was also identified in CRC cell lines by qRT-PCR. **e** Upregulation of ST6GAL1 was detected in CRC tissues. **f** ST6GAL1 expression was higher in advanced CRC patients. **g** High ST6GAL1 expression was shown in CRC samples with metastasis. **h** ST6GAL1 level was detected of CRC cell lines by qRT-PCR. **i** ST6GAL1 expression was also identified in CRC cell lines by western blot. **j** The Kaplan-Meier overall survival curves (OS) was illustrated on the basis of HOTAIR and ST6GAL1 expression. Data were the means ± SD of triplicate determinants (*P < 0.05)

HOTAIR expression was firstly detected by qRT-PCR. As shown in Fig. 1a, higher HOTAIR level was determined in CRC tissues than the corresponding adjacent tissues. Upregulated HOTAIR was detected in the advanced CRC stages (stage III + IV, Fig. 1b). CRC patients with regional or distant metastasis showed higher HOTAIR (Fig. 1c). HOTAIR level was also measured in CRC cell lines by qRT-PCR (Fig. 1d). Interestingly, *ST6GAL1* was confirmed extremely higher in CRC tumor tissues (Fig. 1e). *ST6GAL1* also showed higher level in stage III + IV (Fig. 1f) and patients with metastasis (Fig. 1g). Furthermore, we also measured *ST6GAL1* expression by qRT-PCR (Fig. 1h) and western blot (Fig. 1i) in CRC cell lines. In accordance with the clinical characteristics, upregulated HOTAIR indicated poor CRC prognosis (Fig. 1j). Surprisingly, CRC patients with high *ST6GAL1* predicted poorer prognosis than the patients with low *ST6GAL1* expression. Therefore, high HOTAIR and *ST6GAL1* levels were verified and showed closely correlation with CRC prognosis.

### ST6GAL1 mediates the malignancy of CRC cell lines

**Fig. 2:**
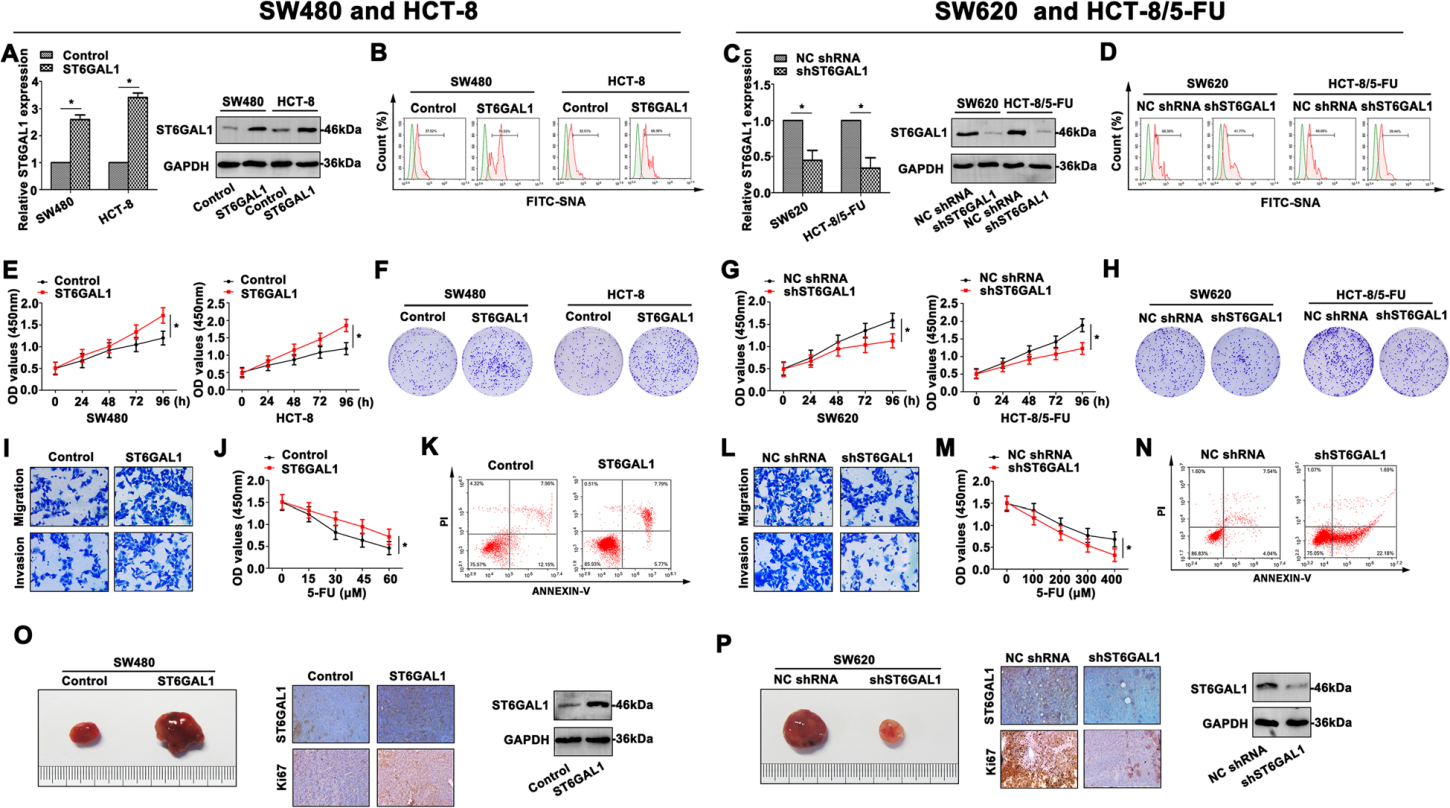
ST6GAL1 mediates the malignancy of CRC cell lines. **a**, **c** The ST6GAL1 expression was detected by qRT-PCR and western blot in the transfected CRC cells. **b**, **d** The FITC-SNA intensity was analyzed by flow cytometry. **e**, **g** The viability of transfected CRC cell lines was examined by CCK8 assay at 0, 24, 48, 72, and 96 h. **f**, **h** Colony formation assay was conducted to measure the proliferation of transfected CRC cell lines. **i**, **l** The migration and invasion of transfected CRC cells were determined by transwell assays. **j**, **m** CCK8 assays were conducted to detect the resistance to 5-FU. The transfected HCT-8/5-FU was treated with different concentration of 5-FU, and the absorbance was measured at 450 nm. **k**, **n** The apoptotic rate was determined by staining Annexin V and PI in the transfected HCT-8/5-FU cells. **o**, **p** The tumorigenesis of transfected CRC cells was shown. IHC staining showed differential ST6GAL1 and Ki67 expression, and differential ST6GAL1 level was also shown by western blot analysis. Data were the means ± SD of triplicate determinants (*P < 0.05)

To further investigate the involvement of *ST6GAL1* in the CRC progression, *ST6GAL1* was manipulated in this study. By qRT-PCR and western blot assays, the ST6GAL1 expression was confirmed in the transfected CRC cell lines (Fig. 2a, c). Lectin on the cell surface was detected to reflect *N*-glycan abundance during cancer development. *Sambucus nigra* (SNA) was used to assess terminal sialic acids attached to penultimate galactose via α 2, 6 linkage of CRC cells. Upregulated ST6GAL1 in SW480 and HCT-8 cells facilitated the SNA intensity (Fig. 2b), however, decreased ST6GAL1 attenuated SNA intensity of the CRC cell surface (Fig. 2d). Overexpressed ST6GAL1 also promoted the proliferation of SW480 and HCT-8 cells by CCK8 assays (Fig. 2e) and colony formation assays (Fig. 2f). On the contrary, shST6GAL1 attenuated the cell growth of SW620 and HCT-8/5-FU cells (Fig. 2g, h). Enhanced aggressiveness of SW480 cells was detected by transwell (Fig. 2i), while *shST6GAL1* in SW620 cells showed a more inhibitory effect on aggressiveness than the control cells (Fig. 2l). Moreover, the chemoresistance to 5-FU was measured by CCK8 assays in the transfected HCT-8 and HCT-8/−FU cells (Fig. 2j, m). The apoptotic rate was detected in response to 5-FU in HCT-8 (Fig. 2k) and HCT-8/5-FU cells (Fig. 2n). The tumorigenesis in nude mice injected with transfected SW480 cells was detected. SW480 cells transfected with ST6GAL1 showed larger tumor volume compared with the control group (Fig. 2o). Higher ST6GAL1 and Ki67 expression were determined by IHC staining. However, SW620 cells transfected with shST6GAL1 showed opposite tendency in tumorigenesis (Fig. 2p). Downregulated ST6GAL1 and Ki67 levels were also confirmed by IHC staining. These data indicated the oncogenic role of ST6GAL1 in CRC proliferation, metastasis and chemoresistance.

### *ST6GAL1* and HOTAIR function as direct target of miR-214

**Fig. 3:**
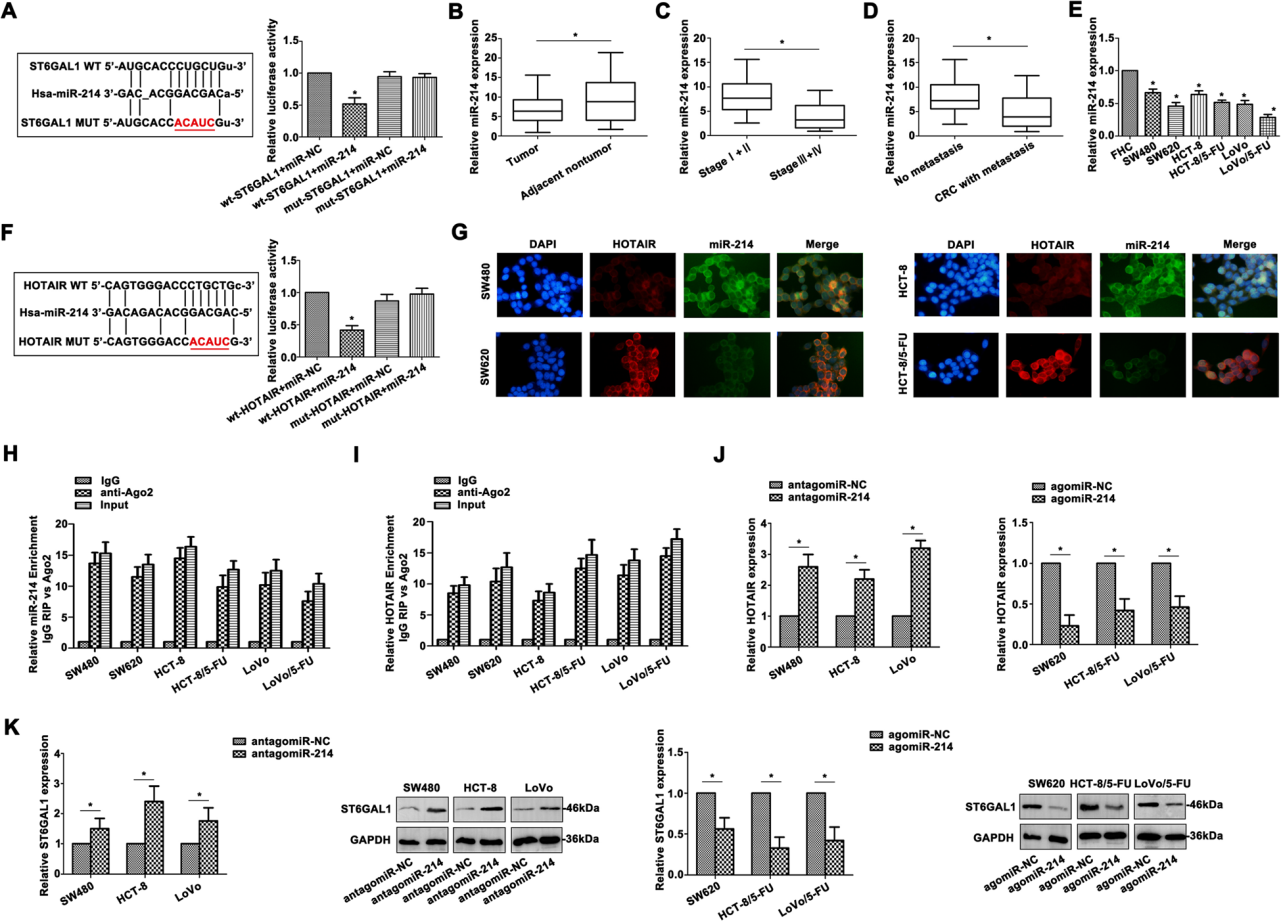
ST6GAL1 and HOTAIR function as direct target of miR-214. **a** The predicted binding sites between ST6GAL1 and miR-214, and the decreased luciferease was also shown to determine the direct binding of ST6GAL1 and miR-214. **b** The miR-214 expression was shown in CRC tissues. **c** Decreased miR-214 was detected in advanced CRC stages (III + IV). **d** CRC patients with metastasis showed decreased miR-214. **e** MiR-214 level was verified in CRC cells by qRT-PCR. **f** The predicted binding sites between HOTAIR and miR-214 were presented, and the dual-reporter luciferase assay confirmed HOTAIR was the direct target of miR-214. **g** The co-location of HOTAIR and miR-214 was verified by FISH assay in CRC cells. **h** The co-precipitated RNA was detected by RNA immunoprecipitation assay. MiR-214 was presented as fold enrichment in Ago2 relative to IgG immunoprecipitate. **i** Enriched HOTAIR was shown by RIP assay. **j** Altered HOTAIR level was detected with the alteration of miR-214. **k** The ST6GAL1 mRNA and protein levels were also determined by qRT-PCR and western blot. Data were the means ± SD of triplicate determinants (*P < 0.05)

To clarify the underlying mechanism that *ST6GAL1* and HOTAIR involved in the CRC, the modulator between *ST6GAL1* and HOTAIR was explored. The binding sites between ST6GAL1 and miR-214 were predicted by bioinformatic analysis. *ST6GAL1* was the direct target of miR-214 by dual-luciferase reporter gene assay (Fig. 3a). Decreased miR-214 was observed in CRC tumor tissues compared to the non-tumor tissues (Fig. 3b). Advanced stages of CRC patients also showed lower miR-214 expression (Fig. 3c). MiR-214 was upregulated in CRC patients without metastasis (Fig. 3d). High metastatic and 5-FU resistant CRC cells depicted lower miR-214 level (Fig. 3e). The binding sites between HOTAIR and miR-214 were presented in Fig. 3f, and HOTAIR was also a direct target of miR-214. Co-location of HOTAIR and miR-214 rendered a possible cross-talk in CRC cells by FISH (Fig. 3g). The enrichment of miR-214 (Fig. 3h) and HOTAIR (Fig. 3i) in Ago2 protein pellet were determined by RIP assays, identifying the potential association between HOTAIR and miR-214. Alteration of miR-214 mediated HOTAIR expression (Fig. 3j). In addition, miR-214 also impacted *ST6GAL1* mRNA and protein levels (Fig. 3k). These data confirmed that miR-214 was a critical modulator between *ST6GAL1* and HOTAIR.

### HOTAIR/miR-214/*ST6GAL1* axis mediates α 2, 6-sialylation of c-met and activates JAK2/STAT3 pathway

**Fig. 4:**
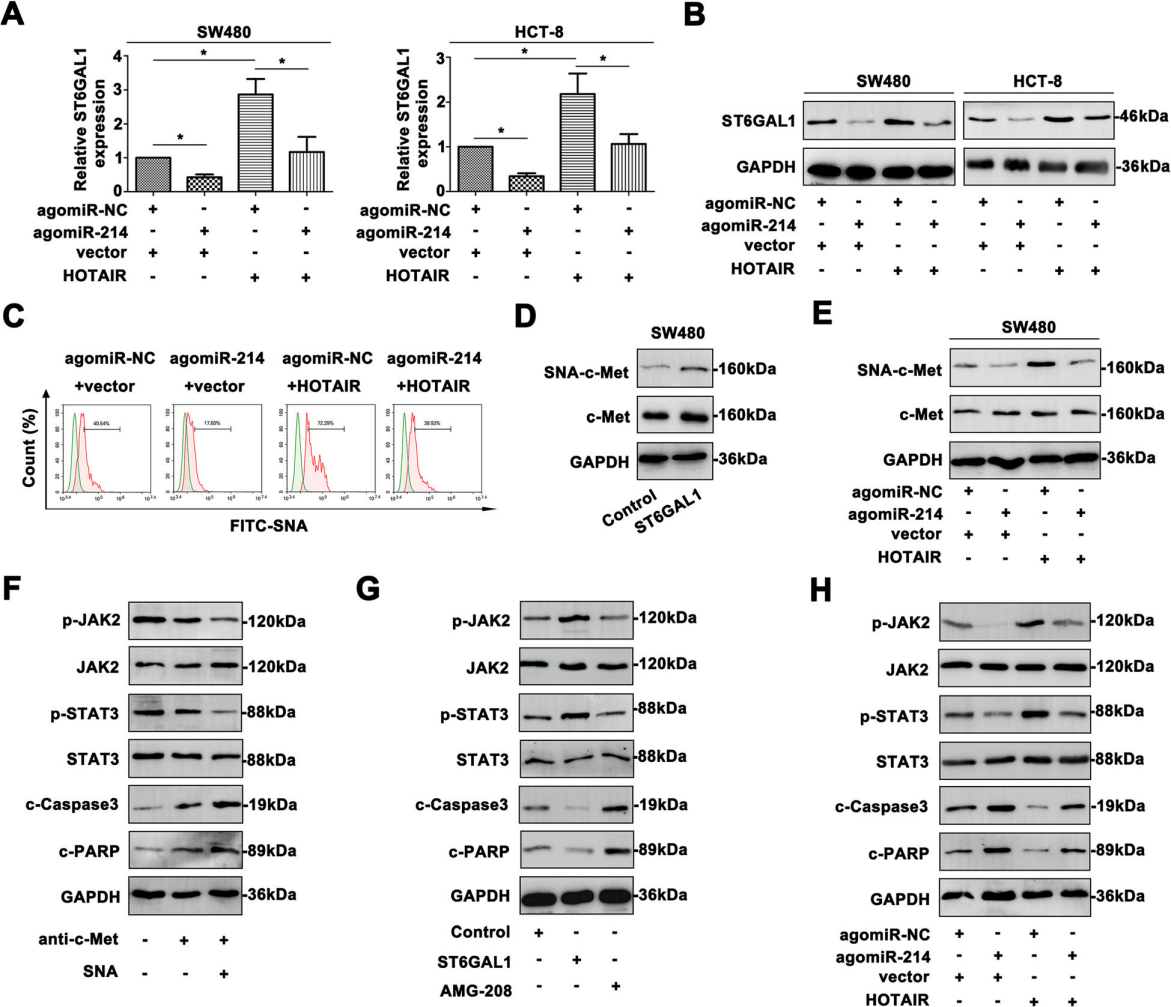
HOTAIR/miR-214/ST6GAL1 axis mediates α 2, 6-sialylation of c-Met and activates JAK2/STAT3 pathway**. a** The ST6GAL1 level was examined by qRT-PCR in CRC cells transfected with HOTAIR or agomiR-214. **b** ST6GAL1 protein level was determined by western blot. **c** Altered FITC-SNA intensity was detected by flow cytometry of transfected cell lines. **d** Upregulated ST6GAL1 facilitated the SNA-binding c-Met expression of SW480 cells. **e** Modulation of HOTAIR and miR-214 affected SNA-binding c-Met level. **f** Activity of JAK2/STAT3 pathway with the treatment of c-Met antibody and SNA was measured. **g** The altered JAK2/STAT3 molecules with the treatment of ST6GAL1 or AMG-208. **h** Alteration of HOTAIR and miR-214 impacted the activity of JAK2/STAT3 signaling. Data were means ± SD of three independent assays (*P < 0.05)

The intrinsic mechanism that HOTAIR and miR-214 regulated *ST6GAL1* in CRC progression was further expounded. The modulatory effect of upregulated miR-214 and HOTAIR on *ST6GAL1* was clarified (Fig. 4a). In comparison to the control group, agomiR-214 inhibited *ST6GAL1* expression, while HOTAIR facilitated *ST6GAL1* level. Co-transfection of agomiR-214 and HOTAIR reversed the *ST6GAL1* expression partially. Similar tendency was revealed by western blot (Fig. 4b). The reversal effect of miR-214 and HOTAIR on *ST6GAL1* expression was expounded, and miR-214 might function as a critical modulator between HOTAIR and *ST6GAL1*. α 2, 6 linked *N*-glycans were altered on the transfected SW480 cell surface by measuring FITC-SNA intensity (Fig. 4c). Highly SNA activity of transfected HOTAIR CRC cells was reversed by co-transfecting agomiR-214. SNA-precipitated c-Met level was measured to indicate the alteration of α 2, 6-sialylated c-Met (Fig. 4d). ST6GAL1 enhanced SNA-precipitated c-Met, while total c-Met was unchangeable. AgomiR-214 attenuated sialylated c-Met degree, and HOTAIR promoted the sialylated c-Met synthesis (Fig. 4e). Co-transfection of agomiR-214 and HOTAIR showed reversal effect on c-Met sialylation. Inactivation of JAK2/STAT3 pathway was measured by anti-c-Met antibody blocking assay and adding SNA, indicating that sialylated c-Met might be a trigger for activating the pathway (Fig. 4f). Moreover, ST6GAL1 strengthened JAK2/STAT3 pathway activation (Fig. 4g). AMG-208, a novel c-Met inhibitor, weakened the activity of JAK2/STAT3 pathway. ST6GAL1 could modulate JAK2/STAT3 pathway by impacting α 2, 6 sialylated c-Met in CRC cells. Additionally, the activity of JAK2/STAT3 pathway was mediated by co-transfecting with miR-214 and HOTAIR in CRC cells (Fig. 4h).

**Fig. 5:**
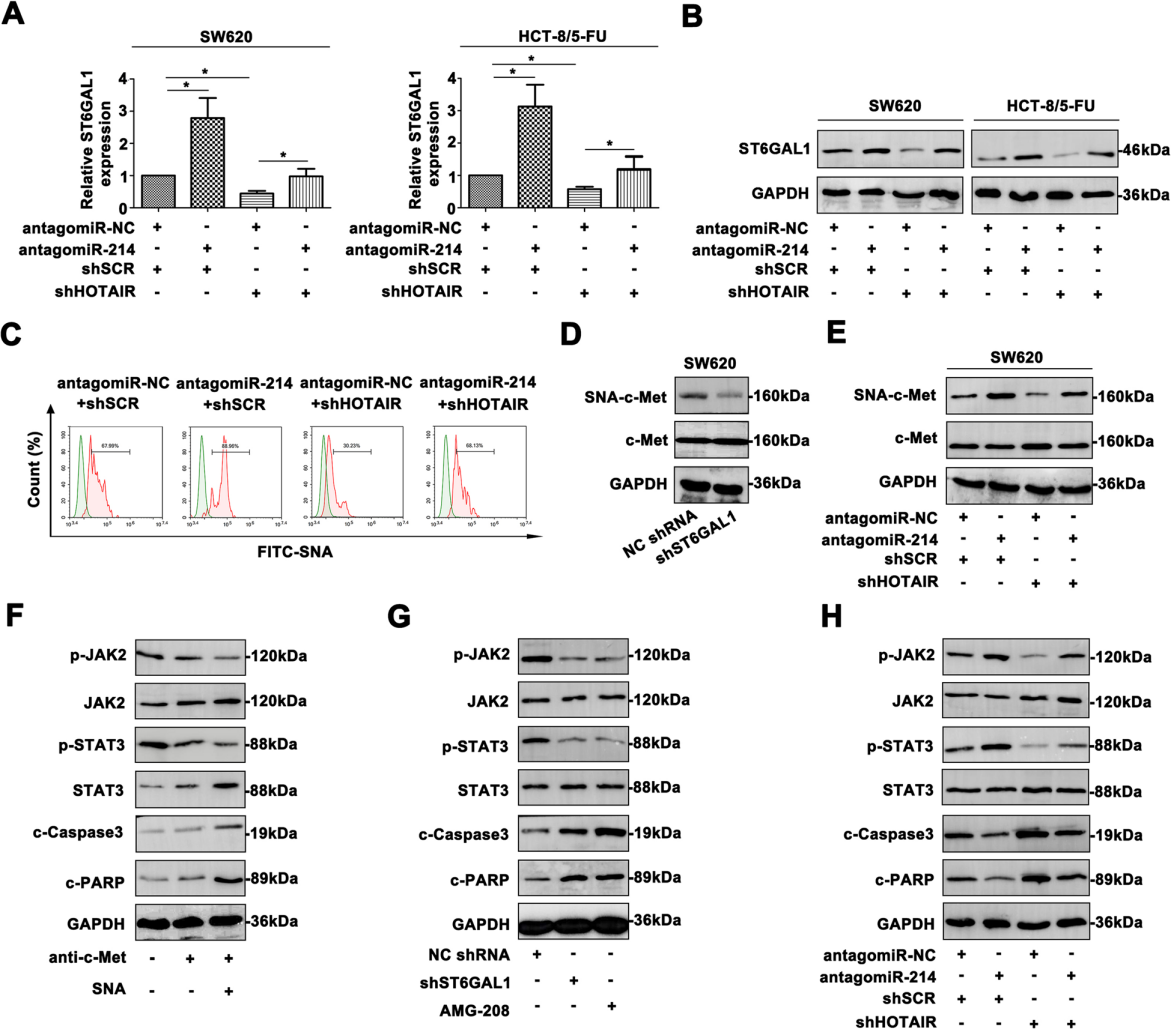
Inhibition of HOTAIR/miR-214/ST6GAL1 axis attenuates α 2, 6-sialylated c-Met during CRC procession and activates JAK2/STAT3 pathway. **a** ST6GAL1 mRNA level was analyzed with antagomiR-214 or shHOTAIR treatment. **b** ST6GAL1 protein level was measured of transfected CRC cells. **c** The FITC-SNA intensity was examined by flow cytometry. **d** Decreased ST6GAL1 attenuated the sialylated c-Met level. **e** Modulation of HOTAIR and miR-214 affected sialylated c-Met level. **f** By blocking c-Met and SNA, the JAK2/STAT3 pathway was inhibited. **g** Decreased ST6GAL1 and AMG-208 inactivated the JAK2/STAT3 pathway. **h** Alteration of shHOTAIR and antagomiR-214 impacted the activity of JAK2/STAT3 signaling. Data were means ± SD of three independent assays (*P < 0.05)

AntagomiR-214 and shHOTAIR in CRC cells also depicted regulatory effect on *ST6GAL1* expression (Fig. 5a, Fig. 5b). Furthermore, FITC-SNA intensity confirmed the altered α 2, 6 sialylation in SW620 cells (Fig. 5c). By SNA binding assay, SNA-precipitated c-Met level was decreased in the cells transfected with shST6GAL1 (Fig. 5d), while total c-Met level was unchangeable. AntagomiR-214 and shHOTAIR affected the sialylated c-Met in CRC cells (Fig. 5e). Sialylation of c-met was declared as the critical trigger for JAK2/STAT3 pathway by antibody blocking assay (Fig. 5f). Knockdown *ST6GAL1* revealed inactivation of the pathway in SW620 cells (Fig. 5g). Moreover, AMG-208 efficiently repressed the JAK2/STAT3 pathway. AntagomiR-214 and shHOTAIR showed regulatory role of the pathway (Fig. 5h). These data provided credible evidence that HOTAIR/miR-214/*ST6GAL1* cross-talk modified the sialylation of c-Met, which indeed triggered JAK2/STAT3 pathway in CRC cells.

### Modulation of HOTAIR and miR-214 influences CRC procession in vitro

**Fig. 6:**
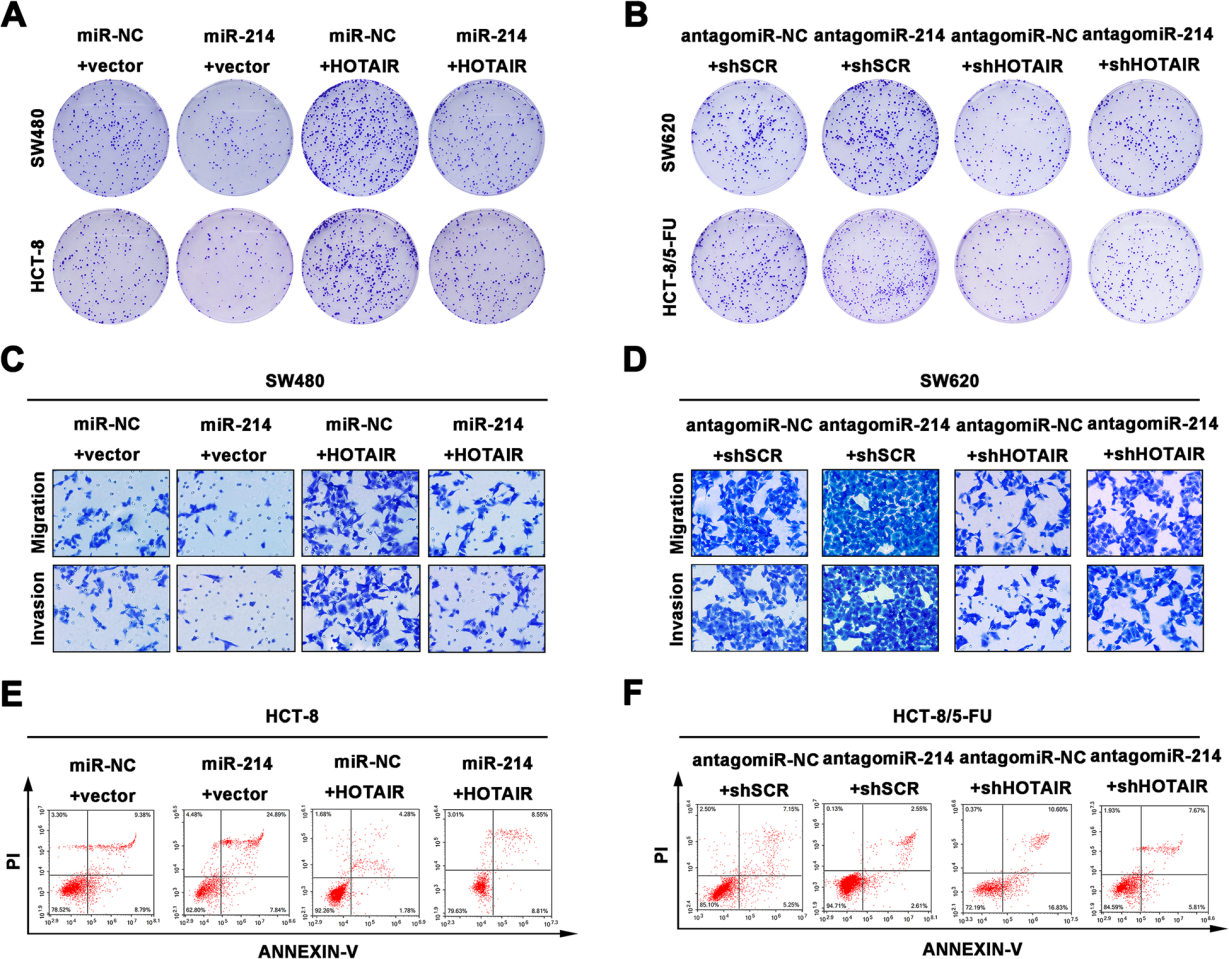
Modulation of HOTAIR and miR-214 influences CRC procession in vitro. (**a**, **b**) Colony formation assay showed the altered proliferative ability of transfected CRC cells. (**c**, **d**) The migration and invasion were measured by transwell assay. (**e**, **f**) The apoptotic rate was detected by flow cytometery. Data were means ± SD of three independent assays (*P < 0.05)

To declare the modulation of HOTAIR and miR-214 on α 2, 6 sialylated c-Met during CRC progression, the functional experiments were further carried out. The proliferation was detected by colony formation assay in the transfected CRC cells. As shown in Fig. 6a, cells transfected with miR-214 showed lower colony number compared to the control group, while cells transfected HOTAIR revealed high colony formation ability. Interestingly, co-transfection miR-214 and HOTAIR reversed the altered proliferation than the cells transfected HOTAIR only. Moreover, knockdown miR-214 or HOTAIR also depicted different proliferation in SW620 and HCT-8/5-FU cells (Fig. 6b). To illustrate the aggressiveness of the transfected SW480 and SW620 cells, transwell assay was carried out. As shown in Fig. 6c, SW480 obtained less invasiveness by transfecting miR-214, while the enhanced migratory and invasive ability were shown in the cells transfected with HOTAIR. MiR-214 was confirmed partly reversed the aggressiveness by co-transfecting of HOTAIR and miR-214 in SW480 cells. The comparable reversal data were shown in transfected SW620 cells (Fig. 6d). We further measured the apoptosis by flow cytometry. As shown in Fig. 6e, HCT-8 cells transfected with miR-214 showed more sensitive to 5-FU, while the cells performed increasingly resistance with upregulated HOTAIR. HCT-8 cells co-transfected with miR-214 and HOTAIR revealed a moderate endurance to 5-FU. As shown in Fig. 6f, co-transfection of antagomiR-214 and shHOTAIR also influenced the apoptosis of HCT-8/5-FU cells. These data provided credible evidence that altered miR-214 and HOTAIR indeed impacted CRC progression in vitro.

### HOTAIR mediates CRC malignancy in vivo

**Fig. 7:**
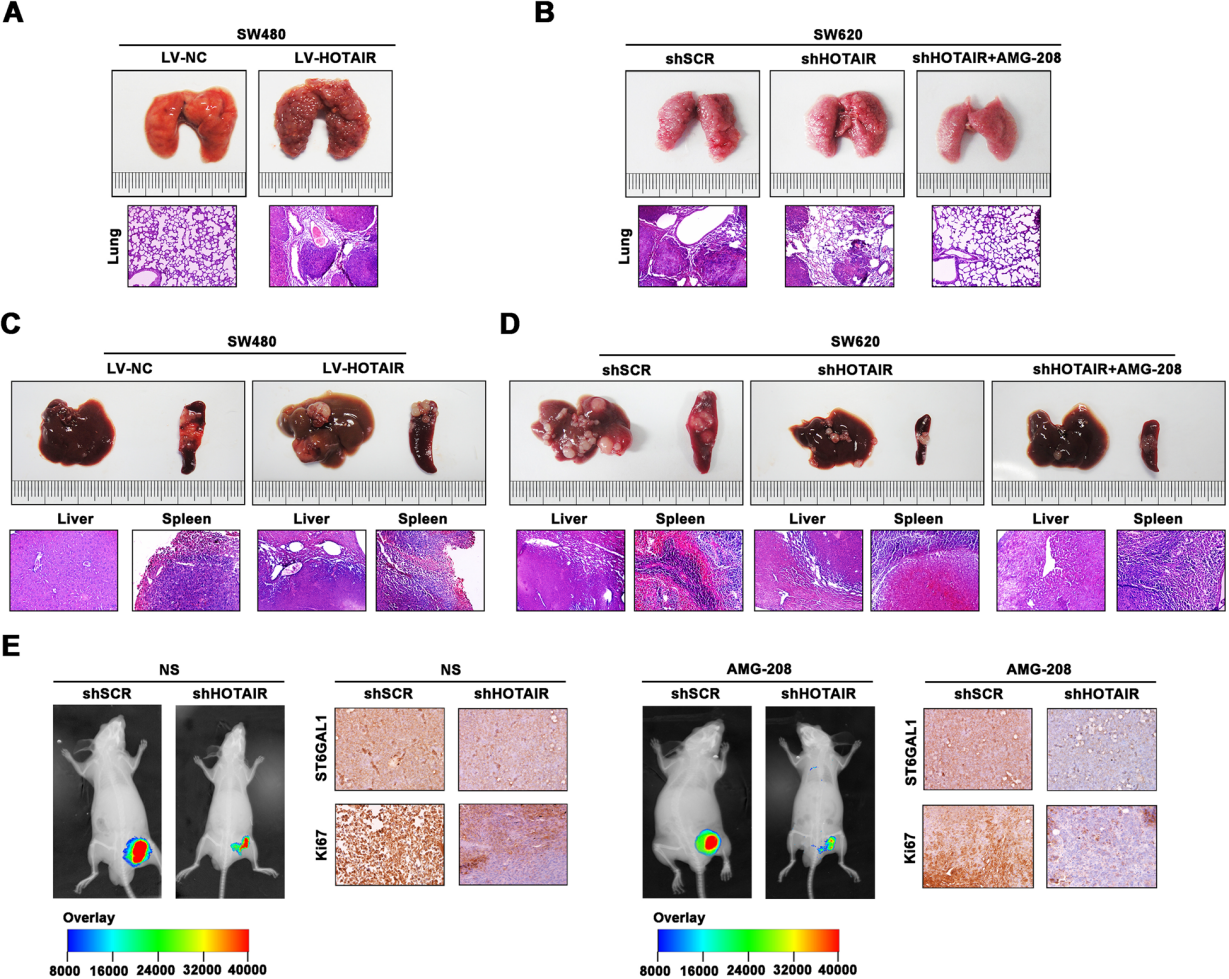
HOTAIR mediates metastasis and tumorigenesis of CRC cells in vivo. **a** The lung metastasis degree of transfected SW480 cells was assessed (up panel). HE staining was presented (down panel). **b** Lung metastasis model was established by SW620 cells transfected with shHOTAIR. The lung metastasis was inhibited by adding AMG-208. **c** The liver metastasis was established with the cells transfected with LV-HOTAIR or LV-NC. **d** CRC liver metastasis model were built with transfected SW620 cells (up panel), and HE staining was presented (down panel). **e** GFP signals of xenograft tumors were obtained with NS and AMG-208. ST6GAL1 and Ki67 levels were measured by IHC staining. Data were means ± SD of three independent assays (*P < 0.05)

To evaluate the regulatory role of HOTAIR in CRC metastasis, the nude mice model were built in vivo. The nude mice bearing HOTAIR transfected SW480 cells showed advanced lung metastasis (Fig. 7a). On the contrary, decreased HOTAIR attenuated the lung metastasis degree (Fig. 7b). In addition, HOTAIR facilitated the liver metastasis degree of SW480 cells (Fig. 7c). Knockdown of HOTAIR suppressed liver metastasis, which was further repressed by adding AMG-208 (Fig. 7d). The inhibitory effect of shHOTAIR on tumorigenesis was verified (Fig. 7e). The tumor volume was smaller in the mice treated with shHOTAIR compared to control group. Moreover, tumor was more repressed in shHOTAIR combined with AMG-208 group. ST6GAL1 and Ki67 levels were evaluated by IHC staining. These data provided promising therapeutic targets for CRC malignancy.

## Discussion

The occurrence of CRC remains a multilevel event, and unrestrained metastasis often led to the advanced CRC progression. Moreover, drug resistance is the main cause of the unsatisfactory therapy. It is imperative to clarify the involvement of modulatory ncRNAs in regulating sialylation during CRC evolution. This study provides in-depth understanding of HOTAIR/miR-214/*ST6GAL1* cross-talk regulating c-Met sialylation via JAK2/STAT3 pathway during CRC progression.

Sialylation exists as a key modification of proteins in cancer. Abnormal sialylation is regarded as the hallmark of tumor development. With the usage of Mass Spectrometry, higher ST6GAL1 is confirmed in CRC cells [20]. Moreover, differential ST6GAL1 is also closely correlated with CRC progression [21]. Similar observations are shown in our study, overexpressed *ST6GAL1* is verified in CRC tissues, and exhibited high association with CRC advancement. Highly *ST6GAL1* level is also verified in CRC cells. Related research depicts the effect of *ST6GAL1* on CRC malignancy [21], and the potential role of *ST6GAL1* in promoting tumor development is also clarified in breast cancer [22]. The critical function of *ST6GAL1* involved into CRC progression is further corroborated and expounded both in vitro and in vivo in this study. ST6GAL1 regulation is found to be the essential factor for CRC malignancy. Therefore, *ST6GAL1* emerges as a promising marker for assessing CRC progression.

Aberrant lncRNA expression is crucial issue for cancer development. High expression of HOTAIR reveals closely association with cancers, such as gastric cancer [13], lung cancer [23] and CRC [20]. Our results provide comprehensive evidence that HOTAIR is extremely associated with poor CRC advancement. For clinical investigation, decreased HOTAIR indicates longer 5-year survival rate. HOTAIR exhibits closely association with CRC malignancy, while the precise mechanism of HOTAIR involved in CRC progression is still unclear. CeRNA theory seems as a reasonable hypothesis to verify the potential mechanism. Reasonably, HOTAIR contributes to CRC liver metastasis by sponging miR-218 [15]. The correlation of HOTAIR and miR-214 is fully expounded in ovarian cancer [19]. By directly binding with *FGFR1*, miR-214 efficiently mediates the procession of CRC liver metastasis [24]. MiR-214 is verified as tumor suppressor of colon cancer [25]. Interestingly, miR-214 also impacts monocyte apoptosis by targeting *PTEN* [26]. In this research, miR-214 is identified as the target of ST6GAL1 and HOTAIR. HOTAIR and miR-214 co-locate in the cytoplasm of CRC cells, which provide the possible interaction between the two molecules. We also affirm the endogenous interaction between HOTAIR and miR-214 as the potential reason for CRC procession. Therefore, HOTAIR/miR-214/*ST6GAL1* crosstalk might efficiently contribute to CRC progression.

Glycoprotein functions as the actual executor of cell physiological and pathological processes. ST6GAL1 is regarded as the essential enzyme to form α 2, 6-linked sialic acids, the major structure of sialylated glycoprotein. Unusual activation of c-Met might be considered as the initiation of cancer malignancy [27]. Aberrant modification of c-Met, with α 2, 6-sialic terminal structure, participates in CRC progression, and often leads to the long-term changes in neoplastic cell motility [8]. In accordance with our study, regulation of miR-214 and HOTAIR results in the altered *ST6GAL1* level. Additionally, ST6GAL1 induces continuous sialylated c-Met activation, which is the potential mechanism accounting for CRC evolution. We hypothesize that HOTAIR and miR-214 could efficiently regulate *ST6GAL1* mRNA level, which induced the alteration of ST6GAL1 protein. The ST6GAL1 enzyme catalyzes the sialylation of c-Met, forms a regulatory HOTAIR/miR-214/*ST6GAL1* crosstalk in commanding α 2, 6 sialylation of c-Met during CRC procession. Recent researches show that c-Met influences the STAT3 dependent pathway [28]. C-Met blocking assay offers us the notion that the directly activation of JAK2/STAT3 pathway is attributed to α 2, 6 sialylated c-Met expression. Moreover, miR-214 also involves in the activation of NF-κB, Wnt, JAK/STAT and TP53 pathways [29]. Co-transfection of HOTAIR and miR-214 also regulates the main molecules of JAK2/STAT3 pathway. HOTAIR and miR-214 influences CRC malignancy. In accordance with the in vitro experimental data, alteration of HOTAIR affects CRC progression, especially the lung and liver metastasis in the nude mouse model. The inhibitory effect of HOTAIR on CRC tumorigenesis is also demonstrated in vivo*.*

The comprehensive research data provide strengthen evidence that HOTAIR/miR-214/*ST6GAL1* axis commands the sialylated c-Met, and further activates JAK2/STAT3 pathway in CRC progression. The HOTAIR/miR-214/*ST6GAL1* crosstalk could be regarded as a potential strategy for CRC. However, CRC is still multi-factorial disease and should be urgent captured. Further investigation is still urgent required.

## Conclusions

The co-mediation of HOTAIR and miR-214 involves in regulation the sialylated c-Met via ST6GAL1, and is clarified in CRC progression. The regulatory network might be inspired for postponing CRC malignancy. The current study presents valuable information regarding the early diagnosis and therapeutic target for CRC.

## Data Availability

Our data, related material and experimental methods were available from the authors based on reasonable request.

## Abbreviations

3’UTR 3′: Untranslated region
AGO2: Argonaute 2
ceRNA: Competing endogenous RNA
CRC: Colorectal cancer
LncRNAs: Long non-coding RNAs
miRNAs: MicroRNAs
OS: Overall survival
RIP: RNA immunoprecipitation
ST6GAL1: α 2, 6-sialyltransferase 1
